# Orthopedic management of pubic symphysis osteomyelitis: a case series

**DOI:** 10.5194/jbji-6-273-2021

**Published:** 2021-07-16

**Authors:** Henry T. Shu, Ahmed H. Elhessy, Janet D. Conway, Arthur L. Burnett, Babar Shafiq

**Affiliations:** 1 Department of Orthopaedic Surgery, Johns Hopkins Hospital, Baltimore, MD, USA; 2 International Center for Limb Lengthening, Rubin Institute for Advanced Orthopaedics, Sinai Hospital, Baltimore, MD, USA; 3 Department of Urology, Johns Hopkins Hospital, Baltimore, MD, USA

## Abstract

**Objectives**: The purpose of this case series is to describe the orthopedic management of pubic symphysis osteomyelitis with an emphasis on the key principles of treating bony infection. Furthermore, we sought to identify whether debridement of the pubic symphysis without subsequent internal fixation would result in pelvic instability. **Methods**: A retrospective chart review was performed to identify all cases of pubic symphysis osteomyelitis treated at both institutions from 2011 to 2020. Objective outcomes collected included infection recurrence, change in pubic symphysis diastasis, sacroiliac (SI) joint diastasis, and ambulatory status. Subjective outcome measures collected included the numeric pain rating scale (NPRS) and the 36-Item Short Form Survey (SF-36). Pubic symphysis diastasis was measured as the distance between the two superior tips of the pubis on a standard anterior–posterior (AP) view of the pelvis. SI joint diastasis was measured bilaterally as the joint space between the ileum and sacrum approximately at the level of the sacral promontory on the inlet view of the pelvis. A paired t test was utilized to compare the differences in outcome measures. An α value of 0.05 was utilized. **Results**: Six patients were identified, of which five were males and one was female (16.7 %), with a mean ± standard deviation (SD) follow-up of 19 ± 12 months (range 6–37 months). Mean ± SD age was 76.2 ± 9.6 years (range 61.0–88.0 years) and body mass index (BMI) was 28.0 ± 2.9 kg/m${}^{2}$ (range 23.0–30.8 kg/m${}^{2}$). When postoperative radiographs were compared to final follow-up radiographs, there were no significant differences in pubic symphysis diastasis (P = 0.221) or SI joint diastasis (right, P = 0.529 and left, P = 0.186). All patients were ambulatory without infection recurrence at final follow-up. Mean improvement for NPRS was 5.6 ± 3.4 (P = 0.020) and mean improvement for SF-36 physical functioning was 53.0 ± 36.8 (P = 0.032). **Conclusion**: This case series highlights our treatment strategy for pubic symphysis osteomyelitis of aggressive local debridement with local antibiotic therapy. Additionally, debridement of the pubic symphysis without subsequent internal fixation did not result in pelvic instability, as determined by pelvic radiographs and ability to fully weight bear postoperatively.

## 1 Introduction

Pubic symphysis osteomyelitis is a rare complication following urological surgery (Kahokehr et al., 2020; Lavien et al., 2017; Nosé et al., 2020). Bony infection of the pubic symphysis following urological surgery typically presents with pain in the groin or paramidline over the pubis with tenderness with or without purulent drainage from previous incisions (Burns and Gregory, 1977; Del Busto et al., 1982; Gupta et al., 2015). Additionally, patients may have difficulty or pain with ambulation over the pubic symphysis (Devlieger et al., 2020). In acute cases of osteomyelitis, there may be elevated C-reactive protein (CRP), leukocytosis, and elevated erythrocyte sedimentation rate (ESR) (Del Busto et al., 1982; Gupta et al., 2015). However, in chronic osteomyelitis, there is a compromised local or systemic immune response, and inflammatory markers may not be elevated (Cierny et al., 2003; Cierny and DiPasquale, 2006; Panteli and Giannoudis, 2016). Imaging, which involves radiographs and magnetic resonance imaging (MRI), may be utilized to further confirm the diagnosis of pubic symphysis osteomyelitis. However, in acute cases of osteomyelitis, standard radiographs may not demonstrate any abnormal findings (Del Busto et al., 1982). In these situations, MRI may be particularly useful; additionally, MRI can identify any urinary fistulas and guide urological surgical planning (Plateau et al., 2015).

One common mechanism by which pubic symphysis osteomyelitis occurs after urological surgery is due to the development of urinary tract fistulas that communicate with the pubic symphysis. The fistulas occur most commonly in males after surgical and/or radiation treatment for prostate malignancy (Becker et al., 2020; Gupta et al., 2015; Kahokehr et al., 2020; Lavien et al., 2017; Minassian et al., 2017; Nosé et al., 2020; Plateau et al., 2015). There are very few reported cases of pubic symphysis osteomyelitis in females, with one case reported as occurring after a Marshall–Marchetti–Krantz procedure for stress urinary incontinence and another after 8 months after vaginal delivery (Burns and Gregory, 1977; Yax and Cheng, 2014). Other causes of pubic symphysis osteomyelitis include hematogenous seeding, pregnancy, and open fracture, which may occur without previous urological surgery (Del Busto et al., 1982; Dudareva et al., 2017; Knoeller et al., 2006).

The key principles of managing osteomyelitis include aggressive debridement of infected tissue, local antibiotic therapy, and dead-space management (Cierny et al., 2003; Cierny and DiPasquale, 2006; Masters et al., 2019; Nandi et al., 2016). Surgical debridement is the cornerstone of treatment as it removes any necrotic, avascular tissue, including involucrum and sequestra and bacterial biofilm (Cierny and DiPasquale, 2006; Swiss orthopaedics and the Swiss Society for Infectious Diseases expert group “Infections of the musculoskeletal system”, 2016). Bacterial biofilms are particularly difficult to eradicate with systemic antibiotics as they create a physical barrier against phagocytic clearance and antimicrobial agents, reduce antibiotic penetrance, and result in a change in bacterial metabolic activity to a more sessile state, which can reduce nutrient dependance and increase resistance to reactive oxygen species (Brady et al., 2008; Masters et al., 2019; Zimmerli and Sendi, 2017). Moreover, because bacterial biofilms have large phenotypic diversity, there is an tendency towards increased antibiotic resistance in the biofilm population via horizontal gene transfer. Some studies have demonstrated that bacteria in a biofilm state can survive antibiotic dosing of up to 1000 times greater than those in their planktonic state (Mah and O'Toole, 2001; Masters et al., 2019; Savage et al., 2013).

In the setting of previous urological surgery, a multidisciplinary approach involving urological surgery is often needed to concomitantly address any urological issues, such as urethral fistulas, while addressing the infected bone. However, previous studies have primarily focused on the urological approach in treating pubic symphysis osteomyelitis, with the orthopedic management of this condition less clear (Gupta et al., 2015; Kahokehr et al., 2020; Lavien et al., 2017; Minassian et al., 2017; Nosé et al., 2020; Plateau et al., 2015). Therefore, the purpose of this case series is to describe the orthopedic management of pubic symphysis osteomyelitis with an emphasis on the key principles of treating bony infection, which include aggressive debridement, local antibiotic therapy, and dead-space management. Furthermore, we sought to identify whether debridement of the pubic symphysis without subsequent internal fixation would result in pelvic instability.

## 2 Methods

All patients who were treated from September of 2011 to September of 2020 for pubic symphysis osteomyelitis at both institutions were included. All included patients were informed that data concerning their cases would be submitted for publication, and all patients provided verbal informed consent.

### 2.1 Surgical technique

For all cases, if there was a need for a urological procedure, the urological portion of the procedure is completed first, before debridement of the pubic symphysis. All patients were positioned supine on the operating room table. Following prepping and draping in a standard sterile fashion, a Pfannenstiel incision is made through the skin (a midline incision can also be used if present from previous procedures) and subcutaneous tissue and hemostasis achieved with electrocautery. Dissection is then carried down through the superficial fascia to the rectus abdominis. The rectus abdominis is then divided in the midline and retracted laterally and anteriorily to access the bladder. The bladder is frequently scarred to the symphysis at the space of Retzius, requiring careful dissection off bone. Dissection to the urethra from this approach is essential to protect it from injury or, more commonly, to identify and repair any urinary fistulas communicating with bone. Repair of the urinary tract is confirmed via cystoscopy and/or an intraoperative dye test. After the urinary tract repair is finished, the bladder and urethra can be protected with a narrow malleable retractor and debridement of the pubic symphysis can be performed safely. The medial tendon of the rectus abdominis is partially elevated off of its insertion on the anterior pubic crest bilaterally and retracted with large Hohmann retractors, similarly to the technique used to plate the pubic symphysis in traumatic disruption. The lateral aspect of the rectus abdominis, which inserts on the pubic tubercles, is preserved. Dissection of the medial rectus abdominis tendon is necessary for resection. Fibrous tissue in the symphysis and parasymphyseal bone is then excised with a rongeur and osteotomes. Resection of parasymphyseal pubic bone is performed until bleeding, healthy bone is reached. Typically this includes 50 % or greater of the parasymphyseal bone but not to the obturator foramen. A bur is utilized to further debride any osteomyelitic bone. At this point, three cultures of bone and symphyseal fibrous tissue are taken and sent for bacterial, fungal, and mycobacterium culture. Once debridement is finished, the defect is copiously irrigated with at least 9 L of normal saline. At the discretion of the attending surgeon, antibiotic cement beads are made from 30 g of polymethylmethacrylate (PMMA) mixed with 4.8 g of tobramycin and 4 g of vancomycin. Once the antibiotic beads are placed, the wound is closed in layers. If antibiotic beads are used, the beads are removed in a second procedure, which is performed approximately 1 week after the initial procedure. The surgical approach for the second surgery utilizes the same incision of the index procedure, antibiotic beads are removed, urinary tract repairs are reinspected to confirm that they are intact, and the joint is debrided and irrigated again. The wound is closed in layers once again. All patients are allowed to weight bear as tolerated postoperatively. Postoperative systemic antibiotic management is dictated by fellowship-trained infectious disease specialists and is based on intraoperative surgical cultures or previously obtained blood cultures and/or urine cultures if surgical cultures are negative. Postoperative antibiotic therapy is continued for at least 6 weeks and extended if the patient demonstrated elevated ESR and CRP on blood work. If intravenous (IV) vancomycin is utilized, therapeutic drug monitoring is used with a goal trough as determined by the Johns Hopkins Hospital Guidelines for Antibiotic Use (Cosgrove et al., 2020). Patient-specific antibiotic therapy is shown in Table 1.

### 2.2 Measurements and statistical analysis

**Figure 1:**
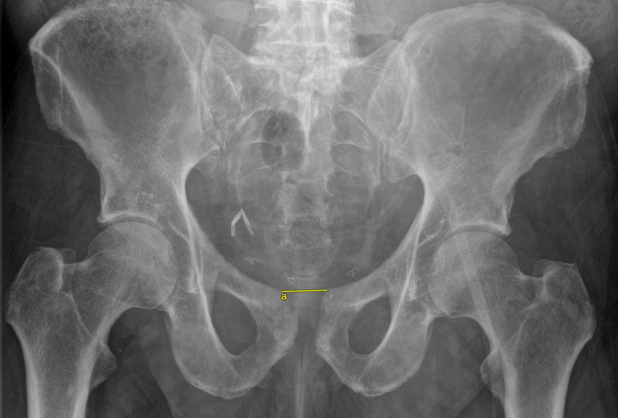
Anterior–posterior radiograph of the pelvis at final follow-up, with the yellow line demonstrating the measurement for pubic symphysis diastasis. The measurement value in this radiograph was 28.6 mm **(a)**.

**Figure 2:**
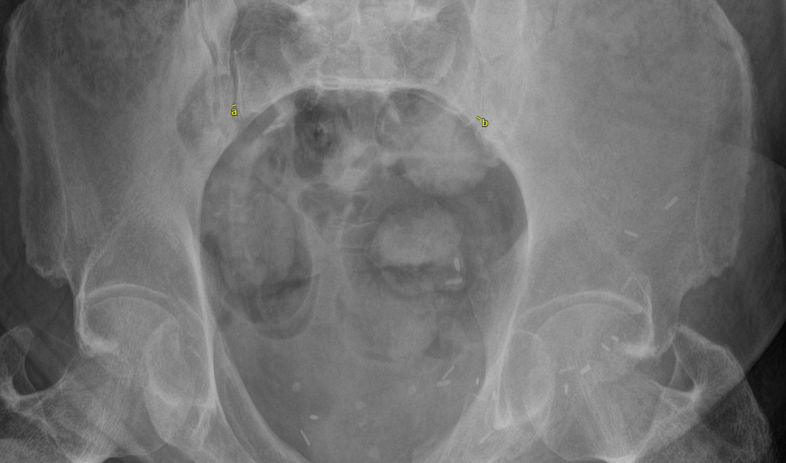
Inlet radiograph of the pelvis, with the yellow lines demonstrating the measurements for sacroiliac (SI) joint diastasis. The measurements were 4.0 mm for the right SI joint **(a)** and 3.7 mm for the left SI joint **(b)**.

Objective outcomes collected included infection recurrence, change in pubic symphysis diastasis, sacroiliac (SI) joint diastasis, and ambulatory status. Subjective outcome measures collected included the numeric pain rating scale (NPRS) and the 36-Item Short Form Survey (SF-36). Pubic symphysis diastasis was measured as the distance between the two superior tips of the pubis on a standard anterior–posterior (AP) view of the pelvis (Fig. 1). SI joint diastasis was measured bilaterally as the joint space between the ileum and sacrum approximately at the level of the sacral promontory on the inlet view of the pelvis (Fig. 2). For both pubic symphysis diastasis and SI joint diastasis, measurements were made on the immediate postoperative films and the last radiographs taken. The NPRS and SF-36 were collected via standardized questionnaires administered by telephone at final follow-up. Infection recurrence was determined clinically, which involved any continued tenderness over the pubic symphysis and/or the presence of any drainage from the incision site. For those patients in whom recurrent infection was suspected based on clinical presentation, additional lab work was first ordered, which included a complete blood count, a comprehensive metabolic panel including CRP, and ESR. If necessary, an MRI was ordered to identify any residual osteomyelitic tissue. A paired t test was utilized to compare the difference in diastasis immediately postoperatively and at last follow-up. A paired t test was also used to compare differences in subjective outcome scores preoperatively and at last follow-up. An α value of 0.05 was utilized.

## 3 Results

Between September of 2011 and September of 2020, a total of six patients were treated for pubic symphysis osteomyelitis, of which five were males and one was female (16.7 %). Mean ± standard deviation (SD) age was 76.2 ± 9.6 years (range 61.0–88.0 years), and body mass index (BMI) was 28.0 ± 2.9 kg/m${}^{2}$ (range 23.0–30.8 kg/m${}^{2}$). Each of the two institutions involved in this study treated three patients. Four out of five males (80.0 %) had received radiation therapy for the treatment of prostate cancer (Table 1). One male did not have a history of prostate cancer and instead had a history of urethral reconstructive surgery for urethral stricture. The one female identified in this study was diagnosed with idiopathic pubic symphysis osteomyelitis, which was confirmed by a computed tomography (CT)-guided biopsy of the pubic symphysis. All patients had chronic osteomyelitis, as defined by the presence of symptoms of at least 6 weeks. All males had a history of recurrent urinary tract infections (UTIs) prior to the development of pubic symphysis osteomyelitis. All patients presented with tenderness over the pubic symphysis and difficulty with ambulation due to pain. All cases of osteomyelitis were confirmed with plain radiographs and MRI, which also demonstrated any urinary fistulas. All cases of pubic symphysis osteomyelitis were Cierny–Mader type III in a type-B host with both local and systemic compromise (Cierny et al., 2003). Mean ± SD follow-up was 19 ± 12 months (range 6–37 months).

**Table 1:** Patient data.

Patient	Sex	Age at index	Previous	Presenting	Surgical	Surgical	Postoperative	Postoperative systemic	Status at
ID		procedure	urological	symptoms	treatment	cultures	complications	antibiotic therapy	final
		(years)	treatment						follow-up
1	Male	88	Radiation therapy for prostate cancer	Tenderness over the pubic symphysis and pain with ambulation	Resection/debridement of pubic symphysis and placement ofvancomycin- andtobramycin-impregnated PMMA beads	Coagulase-negative *staphylococci*	None	10 weeks of IV vancomycin with goaltrough of 13–17 mcg per mL, oral fluconazole 800 mg loading dose followed by oral fluconazole 400 mg daily for 6 weeks	Symptom free,ambulating well
2	Male	70	Urethral reconstructive surgery for urethral stricture	Tenderness over the pubic symphysis and pain with ambulation	Resection/debridement of pubic symphysis and placement ofvancomycin- andtobramycin-impregnated PMMA beads	*Pseudomonas aeruginosa, Candida albicans*	None	6 weeks of oralciprofloxacin 750 mgBID and oral fluconazole 800 mg loading dose followed by oral fluconazole 400 mg daily	Symptom free, ambulating well
3	Male	76	Radiation therapy for prostate cancer	Tenderness over the pubic symphysis and pain with ambulation	Resection/debridement of pubic symphysis and placement of vancomycin- andtobramycin-impregnated PMMA beads	*Enterococcus faecalis*, *Aerococcus urinae*	None	6 weeks of IV piperacillin/tazobactam 3.375 g every 6 h	Symptom free,ambulating well
4	Male	82	Radiation therapy for prostate cancer	Tenderness over the pubic symphysis and pain with ambulation	Resection/debridement of pubic symphysis and placement ofpowdered vancomycin	*Escherichia coli*	None	6 weeks of IV ceftriaxone 2 g daily	Symptom free, ambulating well
5	Female	61	None	Tenderness over the pubic symphysis and pain with ambulation	Resection/debridement of pubic symphysiswithout local antibiotics	No growth	Hematuria at the completion of the case requiring a retrograde cystogram documenting a very small leak between the bladder and the drain. Postoperative wound discharge with positivecultures for vancomycin-resistant *Enterococcus*,successfully managed by wound vacuum and oral antibiotics	6 weeks of IV ceftriaxone 2 g daily and IV clindamycin 600 mg every 8 h. Then startedon oral Sulfamethoxazole/Trimethoprim 800 mg/160 mg BID and oral Metronidazole 500 mg TID for another 6 weeks. At 8 weeks postoperatively, she was also started on oral linezolid 400 mg BID for suspected diverticulitis for another 4 weeks.	Symptom free, ambulating well
6	Male	81	Radiation therapy for prostate cancer	Tenderness over the pubic symphysis and pain with ambulation	Resection/debridement of pubic symphysis and placement ofvancomycin- andtobramycin-impregnated PMMA beads	Methicillin-resistant *Staph aureus*, Group D *Enterococcus*, *Streptococcus viridans*	None	4 weeks of IV linezolid 600 mg BID followed by oral ciprofloxacin 500 mg BID for another 4 weeks	Symptom free,ambulating well

IV: intravenous, BID: bis in die (twice a day), TID: ter in die (three times a day).

All patients had reported previous tobacco use. One patient was a tobacco user at the time of surgery. One patient had previous corticosteroid use as their pubic symphysis osteomyelitis was misdiagnosed as aseptic pubis osteitis. Only one patient had preoperative antibiotic use 2 weeks prior to surgery. He was taking 600 mg of oral cephalexin every 6 h for urinary tract infection prophylaxis.

There was no predominant organism in the surgical cultures (Table 1). Four out of six patients (66.7 %) received vancomycin- and tobramycin-impregnated antibiotic PMMA beads, which were removed at a mean ± SD of 14.3 ± 12.8 d (range 6.0–33.0 d) after the index procedure. One male received only powdered vancomycin at the time of surgery and did not have a second procedure. The one female patient in this study did not receive any local antibiotic therapy. All postoperative systemic antibiotic therapy is described in Table 1. In one male, an incomplete urological repair was identified at the time of antibiotic bead removal, which urology subsequently repaired. The one female included in this study had minor complications postoperatively (Table 1). Her postoperative wound cultures were positive for vancomycin-resistant *Enterococcus*, which was subsequently managed by negative pressure wound therapy and oral trimethoprim/sulfamethoxazole and metronidazole for 6 weeks. She also had an abdominal CT scan 8 weeks postoperatively due to abdominal discomfort, which showed diverticulitis and minimal collection in the pubic area. The patient then completed 4 weeks of linezolid for her diverticulitis. At 12 weeks postoperatively, her ESR and CRP returned to normal, and systemic antibiotic therapy was discontinued. One patient did not have immediate postoperative radiographs stored in our electronic medical records and thus was excluded from radiographic analysis.

When postoperative radiographs were compared to final follow-up radiographs, there were no significant differences in pubic symphysis diastasis (P = 0.221) or SI joint diastasis (right, P = 0.529 and left, P = 0.186) (Table 2). Mean ± SD time between the immediate postoperative radiographs and final follow-up radiographs was 2 ± 1.92 months (range 0.8–5 months). All patients were ambulatory without infection recurrence at final follow-up. Both NPRS and SF-36 subscores were significantly improved following surgery, with the exception of role limitations due to emotional health (Table 3). One patient was not able to complete NPRS or SF-36 surveys as he had died from conditions unrelated to his pubic symphysis osteomyelitis.

**Table 2:** Radiographic outcomes.

	Immediate postoperative	Last	Mean	P value
	radiograph	radiograph	change	
Pubic symphysis diastasis	27.5 ± 16.9	32.5 ± 10.7	5.0 ± 7.7	0.221
Right sacroiliac joint diastasis	3.3 ± 0.8	3.1 ± 0.3	-0.2 ± 0.7	0.529
Left sacroiliac joint diastasis	3.4 ± 0.9	2.8 ± 0.3	-0.6 ± 0.9	0.186

All measurements are in millimeters (mm). All values reported as mean ± standard deviation.

**Table 3:** Subjective outcome measures

	Mean preoperative	Mean last	Mean	P value
	score	follow-up score	change	
NPRS	7.5 ± 3.1	1.9 ± 1.6	-5.6 ± 3.4	0.020
SF-36				
Physical functioning	24.0 ± 40.5	77.0 ± 27.1	53.0 ± 36.8	0.032
Role limitations due to physical health	0.0 ± 0.0	70.0 ± 41.1	70.0 ± 41.1	0.019
Role limitations due to emotional problems	6.7 ± 14.9	53.3 ± 38.0	46.7 ± 38.0	0.052
Energy/fatigue	25.0 ± 16.6	60.0 ± 25.5	35.0 ± 21.8	0.023
Emotional well-being	36.0 ± 29.5	81.6 ± 13.1	45.6 ± 27.2	0.020
Social functioning	35.0 ± 29.8	85.0 ± 27.1	50.0 ± 28.0	0.016
Pain	22.0 ± 20.7	75.5 ± 26.8	53.5 ± 24.3	0.008
General health	39.0 ± 20.7	70.0 ± 5.0	31 ± 18.8	0.021
Health change	0.0 ± 0.0	95.0 ± 11.2	95.0 ± 11.2	< 0.001

NPRS: numerical pain rating score. All values reported as mean ± standard deviation.

## 4 Discussion

The results of this case series suggest that aggressive debridement with local antibiotic therapy and dead space management with antibiotic-impregnated PMMA beads was successful in eradicating pubic symphysis osteomyelitis without evidence of infection recurrence at a mean follow-up of 19 months. As not all patients in this study received local antibiotic therapy or antibiotic-impregnated PMMA beads, aggressive surgical debridement remains the cornerstone of management of chronic osteomyelitis (Cierny and DiPasquale, 2006). This is highlighted by the successful treatment of two patients who had infections with *Candida* species, which are resistant to both vancomycin and tobramycin used in the antibiotic PMMA, and in the one male who received only vancomycin powder with *Escherichia coli*, which is intrinsically resistant to vancomycin (Nikaido, 1989). Effective surgical debridement aims to remove any necrotic tissue and biofilm to restore vascularity and maximize the effectiveness of systemic antibiotic therapy (Brady et al., 2008).

Furthermore, resection of the pubic symphysis without further fixation did not result in pelvic instability, as demonstrated by no significant changes in either pubic symphysis diastasis or SI joint diastasis over the follow-up period. The normal SI joint space has been suggested to be 1.47 ± 0.21 mm on CT in patients without SI joint pain over 40 years of age from the Turkish general population (Demir et al., 2007), which is less than the mean left (2.8 ± 0.3 mm) and right (3.1 ± 0.3 mm) SI joint diastasis as seen in this study. Unsurprisingly, the mean ± SD pubic symphysis diastasis of 32.5 ± 10.7 mm at final follow-up in this study is greater than the average (12.18 ± 12 mm) reported by Alicioglu et al. (2008) in a CT study at a Turkish academic center. This is, of course, attributable to pubic symphysis and parasymphyseal bone resection. The change in diastasis from immediate postoperative radiographs to final radiographs was not statistically significant. The excellent subjective outcomes and ambulatory status of our patients suggest that diastasis after resection does not result in functional deficits.

Additionally, all patients could weight bear as tolerated postoperatively, further suggesting that additional fixation is not necessary for pelvic stability. There was also a significant improvement in the mean NPRS and SF-36 scores, with the largest improvement seen in role limitations due to physical health (70.0 ± 41.1) and general health change (95.0 ± 11.2). Although the minimal clinically important differences (MCIDs) for the SF-36 have not been established for bone infections, the improvements in the SF-36 in our patients surpassed the MCIDs for the SF-36 regarding lower extremity osteoarthritis, hip arthroplasty, and knee arthroplasty (Angst et al., 2001; Jayadevappa et al., 2017; Keurentjes et al., 2012).

As all patients in this study had chronic osteomyelitis when they presented to the orthopedic service, they were considered candidates for surgical intervention as they had failed nonoperative treatment with systemic antibiotics alone. Furthermore, many of these patients had fistulas connecting the pubic symphysis to the urinary tract, which necessitates urological repair to prevent recurrent microbial seeding of the pubic symphysis (Becker et al., 2020). Recently, Becker et al. (2020) have shown that patients with any draining fistulas to the pubic symphysis had a hazard ratio of 5.1 (P=0.011) for treatment failure (Becker et al., 2020). Although not statistically significant, they also show that polymicrobial infections had a hazard ratio of 70.5 (P=0.090) for treatment failure. Thus, in patients with chronic pubic symphysis osteomyelitis with fistulas, we suggest that a multidisciplinary surgical approach should be the treatment of choice for complete resolution of the infection. However, in cases of acute pubic symphysis osteomyelitis, systemic antibiotic therapy may be considered as bony vascularity may still be preserved and biofilm formation may not have occurred (Brady et al., 2008; Zimmerli and Sendi, 2017). There have been reports of successful treatment of acute pubic symphysis osteomyelitis without surgical intervention (Burns and Gregory, 1977; Del Busto et al., 1982; Knoeller et al., 2006; Minassian et al., 2017; Yax and Cheng, 2014).

In this case series, there was large diversity in the species of organisms grown from surgical cultures. Of note, there were both Gram-positive and Gram-negative bacteria and fungal species, primarily of the *Candida* genus. All patients that had positive fungal cultures received oral fluconazole for antifungal coverage. As two out of six of our patients had fungal infections, this case series suggests that clinicians should be aware of possible fungal infection in these cases of osteomyelitis, especially as none of our patients reported antifungal treatment prior to their surgical debridement. Antibiotic management of these patients may be particularly challenging as these patients may have had several courses of empiric antibiotic therapy prior to hospital admission and had numerous positive cultures from blood and urine, which sometimes demonstrated different organisms than surgical cultures. In these chronic infections, previously cultured organisms may no longer be present and antibiotic resistance may be present due to previous courses of antibiotics. Therefore, we suggest that surgical cultures should be the primary guide for postoperative antibiotic therapy (Tiemann and Hofmann, 2009).

There have been very few cases of pubic symphysis osteomyelitis reported in females, and it is suggested to be a rare complication associated with pregnancy and/or delivery (Boyles and Costantine, 2020; Burns and Gregory, 1977; Cosma et al., 2019; Devlieger et al., 2020; Gamble et al., 2006; Yax and Cheng, 2014). In men, pubic symphysis osteomyelitis is more commonly due to the treatment of prostate cancer (Albers et al., 2018; Gupta et al., 2015; Kahokehr et al., 2020; Lavien et al., 2017; Nosé et al., 2020; Plateau et al., 2015). The mechanism by which radiation therapy for prostate cancer has been suggested to cause pubic symphysis osteomyelitis is iatrogenic osteonecrosis of the joint (Minassian et al., 2017). In this present study, four out of five males had received radiation therapy for prostate cancer, further supporting the notion that pubic symphysis osteomyelitis is a rare complication following radiation therapy. Although the female in our study was diagnosed with idiopathic pubic symphysis osteomyelitis, we speculate that she likely acquired it via hematogenous seeding as she had no prior urological procedure or fistulas to her urinary tract.

There is growing interest in the use of antibiotic-loaded resorbable ceramic biocomposites as vehicles for local antibiotic delivery (Ferguson et al., 2017, 2014; McNally et al., 2016). Gamble et al. (2006) reported using a single-stage procedure with calcium phosphate beads loaded with tobramycin and vancomycin to treat a case of female pubic symphysis osteomyelitis that developed in the third trimester of pregnancy (Gamble et al., 2006). We decided to use antibiotic PMMA beads as our vehicle for local antibiotic therapy as it has been demonstrated to be a safe and effective method for the treatment of septic joints and osteomyelitis (Gogia et al., 2009; van Vugt et al., 2019). There is some evidence to suggest that antibiotic resorbable biocomposites can be used to treat infected total joints in a single-stage procedure (Abosala and Ali, 2020; Cowie et al., 2019). Thus, pubic symphysis osteomyelitis could potentially be treated similarly to a single-stage procedure utilizing an antibiotic-loaded resorbable ceramic biocomposite. Complications following the use of ceramic biocomposites include serous wound drainage and hypercalcemia (Kallala and Haddad, 2015; Menon et al., 2018). Furthermore, some authors have reported managing pubic symphysis osteomyelitis nonoperatively with IV and oral antibiotics (Albers et al., 2018; Cosma et al., 2019; Del Busto et al., 1982; Yax and Cheng, 2014). Nevertheless, debridement remains the mainstay of treatment for chronic bony infection given that biofilm formation and bony sequestra may limit immunologic and antibiotic penetration (Masters et al., 2019; Swiss orthopaedics and the Swiss Society for Infectious Diseases expert group “Infections of the musculoskeletal system”, 2016).

### Limitations

This study is primarily limited by its retrospective nature and small sample size. However, the fact that only six cases have been identified across two institutions over the span of 9 years highlights the rarity of pubic symphysis osteomyelitis. This is also the first study to examine pelvic stability following debridement of the pubic symphysis. Although CT scans are considered the most accurate method of measuring pubic symphysis and SI joint space, no pelvis CT scans were performed postoperatively in any of our patients (Alicioglu et al., 2008; Demir et al., 2007). Nonetheless, our measurements have internal validity as we ensured that all measurements were taken in a standardized process.

## 5 Conclusions

This case series highlights our treatment strategy for pubic symphysis osteomyelitis of aggressive local debridement with local antibiotic therapy. Additionally, debridement of the pubic symphysis without subsequent internal fixation did not result in pelvic instability, as determined by pelvic radiographs and ability to fully weight bear postoperatively.

## Data Availability

Identifiable personal data cannot be accessed by the public under the Health Insurance Portability and Accountability Act of the United States.
